# MicroRNA-532-3p Modulates Colorectal Cancer Cell Proliferation and Invasion via Suppression of FOXM1

**DOI:** 10.3390/cancers16173061

**Published:** 2024-09-02

**Authors:** Ketakee Mahajan, Ani V. Das, Suresh K. Alahari, Ramesh Pothuraju, S. Asha Nair

**Affiliations:** 1 Cancer Research Program-4, Rajiv Gandhi Centre for Biotechnology, Thiruvananthapuram 695014, Kerala, India; ketakee@rgcb.res.in (K.M.); anivdas@rgcb.res.in (A.V.D.); rameshp@rgcb.res.in (R.P.); 2 Research Centre, University of Kerala, Thiruvananthapuram 695034, Kerala, India; 3 Department of Biochemistry and Molecular Biology, Louisiana State University Health Sciences Center, New Orleans, LA 70112, USA

**Keywords:** microRNA, colorectal cancer, FOXM1, metastasis

## Abstract

**Simple Summary:**

Forkhead-box M1 (FOXM1) is a proliferation-associated transcription factor, overexpressed in almost all the cancers, making its regulation worth investigating. FOXM1 is known to bind to the promoters of certain microRNAs, a class of biomolecules involved in post-transcriptional gene regulation. Database mining followed by Venn analysis led us to the shortlisting of seven microRNAs, among which microRNA-532-3p showed the strongest interaction with *FOXM1* 3’UTR. The ectopic overexpression of miR-532-3p in colorectal cancer (CRC) cells led to decreased FOXM1 levels which resulted in the alteration of CRC functionalities. These altered phenotypes were supported by a change in the expression of proliferation and EMT markers. In conclusion, the present study suggested that the regulation of *FOXM1* by microRNA-532-3p via its interaction with *FOXM1* 3’UTR inhibited CRC proliferation, migration, and invasion.

**Abstract:**

Colorectal cancer (CRC) is a heterogeneous disease and classified into various subtypes, among which transcriptional alterations result in CRC progression, metastasis, and drug resistance. Forkhead-box M1 (FOXM1) is a proliferation-associated transcription factor which is overexpressed in CRC and the mechanisms of *FOXM1* regulation have been under investigation. Previously, we showed that FOXM1 binds to promoters of certain microRNAs. Database mining led to several microRNAs that might interact with *FOXM1* 3’UTR. The interactions between shortlisted microRNAs and *FOXM1* 3’UTR were quantitated by a dual-luciferase reporter assay. MicroRNA-532-3p interacted with the 3’UTR of the *FOXM1* mRNA transcript most efficiently. MicroRNA-532-3p was ectopically overexpressed in colorectal cancer (CRC) cell lines, leading to reduced transcript and protein levels of *FOXM1* and *cyclin B1*, a direct transcriptional target of FOXM1. Further, a clonogenic assay was conducted in overexpressed miR-532-3p CRC cells that revealed a decline in the ability of cells to form colonies and a reduction in migratory and invading potential. These alterations were reinforced at molecular levels by the altered transcript and protein levels of the conventional EMT markers E-cadherin and vimentin. Overall, this study identifies the regulation of *FOXM1* by microRNA-532-3p via its interaction with *FOXM1* 3’UTR, resulting in the suppression of proliferation, migration, and invasion, suggesting its role as a tumor suppressor in CRC.

## 1. Introduction

Colorectal cancer (CRC) stands as the third most prevalent and second most lethal cancer worldwide. Its occurrence ranks second and third among women and men, respectively [1]. The incidence of CRC has seen a consistent increase over recent decades [2]. The causes of CRC can be either non-hereditary or sporadic, involving the downregulation of tumor suppressor genes and genes responsible for regulating cell cycle processes, DNA damage repair, and apoptosis [3]. Traditionally, CRC has been documented predominantly in the elderly population, affecting individuals aged 50 years and above [4]. The likelihood of developing CRC is notably higher among those with inflammatory bowel diseases (IBDs) such as ulcerative colitis and Crohn’s disease [5].

Forkhead box M1 (FOXM1) stands as a pivotal member within the forkhead box proteins family, a group of transcription factors [6]. Functioning as a master regulator of the cell cycle, FOXM1 plays a crucial role in orchestrating the G2/M transition. It exerts direct control over the expression of key elements such as Aurora B kinase, Cyclin B, and Cdc25b phosphatase [7]. Notably, FOXM1 has demonstrated overexpression across a wide spectrum of cancers [8,9]. Elevated levels of FOXM1 expression have been associated with an unfavorable prognosis in breast cancer, esophageal adenocarcinoma [10], and CRC [11,12]. Beyond its positive regulatory role in the cell cycle, FOXM1 engages in various cancer-related functions such as cellular proliferation, migration, invasion [13], and resistance to drugs [14]. The multifaceted nature of FOXM1 positions it as a potential therapeutic target, sparking interest in suppressing its expression for over a decade [15]. Within the eukaryotic genome, specific regions encode various types of non-coding RNAs, primarily categorized into short and long noncoding RNAs [16]. Noteworthy examples of short noncoding RNAs include microRNAs, piRNAs, and snoRNAs [17,18].

MicroRNAs represent a group of evolutionarily conserved, naturally occurring small nucleotides measuring approximately 19–24 bases in length. Their primary role involves the post-transcriptional regulation of gene expression, accomplished through binding to mRNA transcripts. This interaction disrupts the translational complex, thereby impeding protein synthesis. Beyond translational inhibition, microRNAs are recognized for their ability to destabilize and degrade messenger RNA molecules [18,19].

MicroRNA-532-3p is an upcoming tumor suppressor in various cancers. In CRC, it has been reported to inhibit Wnt/β-catenin signaling, effectively impeding the disease’s progression. The present study corroborates these findings by revealing its ability to enhance cleavage in PARP and Pro-caspase 7. Notably, the ectopic overexpression of miR-532-3p in CRC cells resulted in a significant reduction in proliferative potential, migration, and invasion, corroborated with the previous study [20]. Similarly, its role in suppressing proliferation and facilitating apoptosis has been investigated in lymphoma, where it targets β-catenin [21]. In prostate cancer, miR-532-3p was found to impede bone metastasis by disabling NF-κB signaling [22].

In the present study, we have discussed the role of miR-532-3p in suppressing the cellular proliferation, migration, and invasion of CRC. MicroRNA-532-3p has been reported to act as a tumor suppressor in a renal cell carcinoma [23] and colorectal cancer [21]. Both strands of microRNA-532, viz. miR-532-5p, and miR-532-3p, are known to positively and negatively impact the occurrence and prognosis of several types of cancers such as breast cancer, hepatocellular carcinoma and ovarian cancer [24,25,26,27,28,29].

## 2. Results

### 2.1. MicroRNA-532-3p Interacts with 3’UTR of FOXM1 Transcript

**Figure 1:**
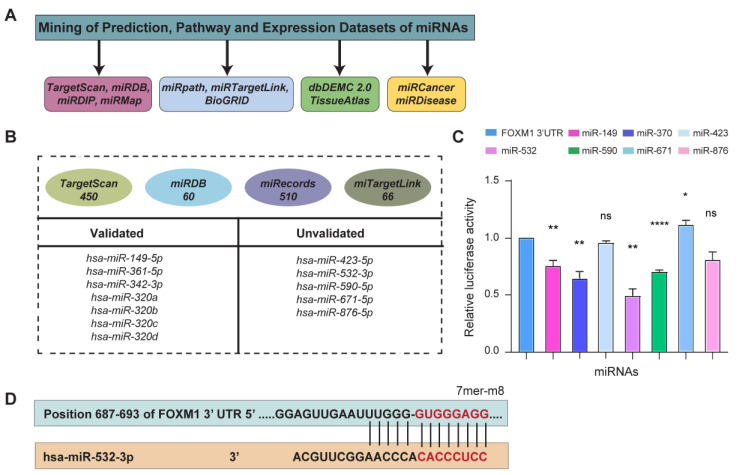
**Selection of candidate microRNA that might bind to *FOXM1* 3’UTR.** (**A**) Mining of prediction, pathway, and expression databases to find microRNAs that might bind to *FOXM1* 3’UTR. (**B**) Analysis of hits obtained from several prediction algorithms. (**C**) Dual-luciferase reporter assay to evaluate the predicted interaction between *FOXM1* 3’UTR and selected microRNAs. (**D**) A prediction of binding between microRNA-532-3p and *FOXM1* 3’UTR. (*: *p* = 0.05, **: *p* < 0.01, ****: *p* < 0.0001).

The database mining revealed several microRNAs potentially capable of regulating the expression by binding through the 3’UTR of the *FOXM1* transcript (Figure 1A). Databases such as TargetScan and miRecords gave more than 450 hits each with possible complementary seed and target sequences in microRNAs and the 3’UTR, respectively. Network databases such as miRtargetLink also predicted several interactions, along with reporting a few validated ones. The microRNAs were analyzed for the most frequently occurring hits by Venn analysis (Figure 1B).

The selected microRNAs, cloned in pRIP vector, were co-transfected with Psicheck2 vector containing 3’UTR of *FOXM1* insert, with a firefly luciferase gene in the downstream region (Figures S1–S3). The dual-luciferase assay confirmed the interactions of miR-149-5p and -3p, miR-370-3p, miR-532-3p, miR-590-5p, miR-671-5p, and miR-876-5p with *FOXM1* 3’UTR, indicated by the reduced luciferase activity. MicroRNA-532-3p showed nearly 50% reduction in luciferase activity, suggesting a strong interaction (Figure 1C). Hence, miR-532-3p was selected for further investigation. A representation of a possible interaction between miR-532-3p and *FOXM1* 3’UTR was obtained from TargetScan database (Figure 1D).

### 2.2. miR-532-3p Is Downregulated in Colorectal Cancer

**Figure 2:**
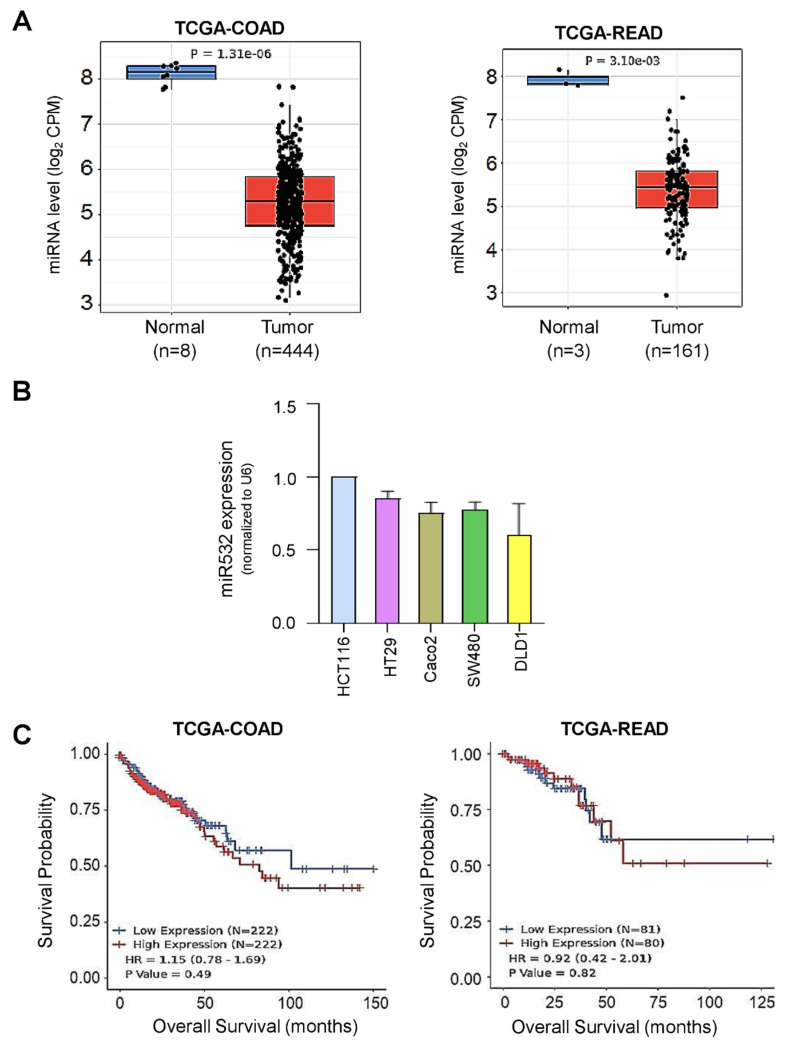
**MicroRNA-532-3p in CRC.** (**A**) Expression of miR-532-3p in tumors against normal tissues of colon and rectum (TCGA). (**B**) Expression of miR-532-3p in CRC cells. (**C**) Survival analyses of colon adenocarcinoma and rectal adenocarcinoma cases against expression levels of miR-532-3p (TCGA).

Investigating The Cancer Genome Atlas (TCGA) datasets revealed that the expression of microRNA-532-3p was significantly lower in colorectal tumors compared to their normal counterparts (Figure 2A). Several CRC cell lines were screened for the expression of microRNA-532-3p, revealing that HCT116 showed the highest expression level (Figure 2B). However, Kaplan–Meier analysis showed no significant decrease in overall survival in colon and rectal patients with high miR-532-3p (Figure 2C).

### 2.3. miR-532-3p Overexpression Diminishes Proliferation, Migration, and Invasion of CRC Cells

**Figure 3:**
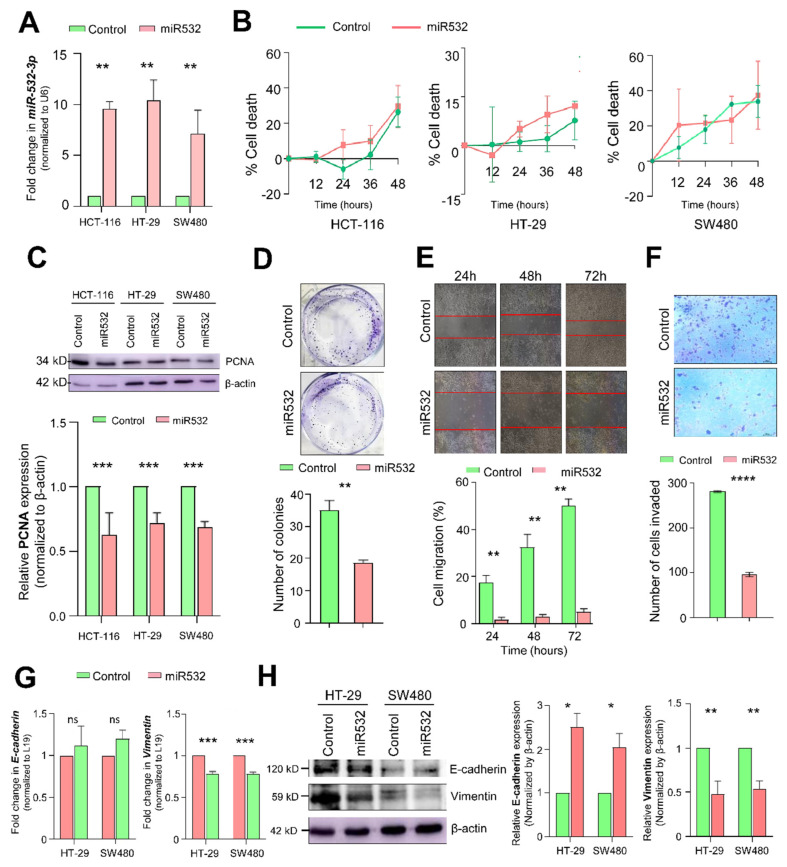
**Effect of miR-532-3p in CRC cells.** (**A**) Ectopic overexpression of microRNA-532-3p in CRC cells (*p* = 0.0012). (**B**) Effect of ectopic overexpression of miR-532-3p on cell viability in HCT116, HT29, and SW480 cells. Effect of ectopic overexpression of miR-532-3p on cellular proliferation by (**C**) expression of PCNA, a cell proliferation marker (*p* = 0.0004) in CRC cells and (**D**) colony formation assay (*p* = 0.0068) in HT29 cells. Effect of ectopic overexpression of miR-532-3p on (**E**) cellular migration (*p* = 0.011) and (**F**) matrigel invasion (*p* < 0.0001) in SW480 cells. Levels of EMT biomarkers E-cadherin and vimentin at (**G**) transcript (*p* = 0.0315, *p* < 0/0001, respectively) and (**H**) protein (*p* = 0.0589, *p* = 0.0038, respectively) level on ectopic overexpression of miR-532-3p. (*: *p* = 0.05, **: *p* < 0.01, ***: *p* < 0.001, ****: *p* < 0.0001).

To understand the functional significance of miR-532-3p, the ectopic overexpression of miR-532-3p in various CRC cell lines was performed (Figure 3A). An evaluation of potential cell death resulting from miR-532-3p overexpression was performed through a colorimetric analysis using the MTT assay (Figure 3B). Intriguingly, no notable disparity in cell viability was observed upon miR-532-3p overexpression. However, a reduction in the expression of the cell proliferation marker PCNA was detected in CRC cell lines overexpressing miR-532-3p (Figure 3C). Furthermore, the ectopic overexpression of miR-532-3p in HT29 cells led to about a 40% reduction in the number of colonies formed (Figure 3D). A wound healing experiment conducted in SW480 cells with miR-532-3p overexpression revealed a slower rate of wound closure in the cells overexpressing miR-532-3p compared to the control at various time points (Figure 3E). Additionally, a significant reduction was noted in the invasion of SW480 cells through matrigel in those ectopically overexpressing miR-532-3p (Figure 3F). These observations were further supported by a quantitative analysis of molecular markers at both transcript and protein levels. The said analyses revealed upregulated levels of the epithelial marker E-cadherin and a reduced expression of vimentin in cells overexpressing miR-532-3p (Figure 3G,H). These findings suggest that miR-532-3p influences cellular proliferation, migration, and invasion properties in CRC *in vitro*.

### 2.4. miR-532-3p Overexpression Led to Higher Apoptosis, Possibly via Suppression of FOXM1

**Figure 4:**
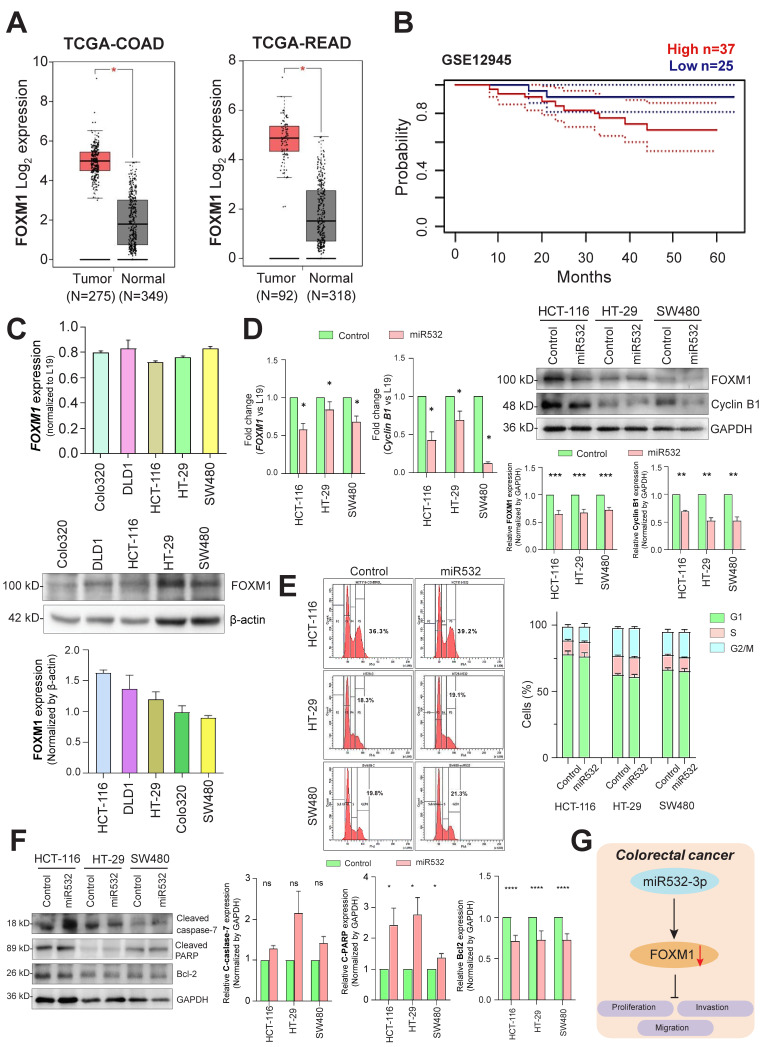
**MicroRNA-532-3p regulates the expression of FOXM1.** (**A**) Relative expression levels of *FOXM1* in colon and rectal adenocarcinoma; tumor vs. normal tissues. (**B**) Overall survival of CRC patients with respect to *FOXM1* expression levels (Dataset GSE12945, Prognoscan). Constitutive expression of *FOXM1* at (**C**) protein and transcript level in CRC cells. (**D**) Effect of ectopic overexpression of miR-532-3p on *FOXM1* expression at transcript (*p* = 0.0185) and protein (*p* = 0.0001) levels and on cyclin B1 protein level (*p* = 0.002) in CRC cells. (**E**) Effect of ectopic overexpression of miR-532-3p on cell cycle progression in CRC cells. (**F**) Effect of ectopic overexpression of miR-532-3p on expression of apoptotic biomarkers cleaved caspase-7 (*p* = 0.0876), cleaved PARP (*p* = 0.0488), and anti-apoptotic marker BCL2 (*p* < 0.0001) in CRC cells. (**G**) MicroRNA-532-3p diminishes the expression of *FOXM1* post-transcriptionally, resulting in suppressed proliferation, migration, and invasion in CRC cells. (*: *p* = 0.05, **: *p* < 0.01, ***: *p* < 0.001, ****: *p* < 0.0001).

Primary findings from the TCGA datasets revealed that the expression levels of *FOXM1* are significantly altered between tumors and their disease-free counterparts in both colon and rectal tissues (Figure 4A). Furthermore, in terms of survival, TCGA revealed no significant difference between the survival rates of patients exhibiting high levels of *FOXM1* against those with low levels (Dataset GSE12945) (Figure 4B). Several CRC cell lines were screened for the expression of *FOXM1* at transcript and protein levels, thereby showing the high transcript levels of *FOXM1* in DLD1 and SW480 cells and high protein level expression in HCT116 and HT29 cells (Figure 4C).

The ectopic overexpression of microRNA-532-3p resulted in reduced transcript and protein levels of *FOXM1*. This confirmed the ability of miR-532-3p to regulate *FOXM1* at the post-transcriptional level. Furthermore, the levels of cyclin B1 were analyzed, which is a direct transcriptional target of FOXM1. A real-time PCR and Western immunoblotting revealed that overexpressing miR-532-3p resulted in reduced transcript and protein levels of cyclin B1 in CRC cells (Figure 4D).

To determine whether the overexpression of miR-532-3p may play a role in cell cycle progression, we analyzed the DNA content by a flow cytometry analysis of CRC cells; overexpressing showed very little increase in the percentage of cells in the G_2_/M phase (Figure 4E). However, Western immunoblotting analysis showed increased levels of several apoptotic markers such as an activated form of caspase 7 and cleaved PARP levels. Additionally, an anti-apoptotic BCL2 marker was found to be downregulated (Figure 4F).

In essence, microRNA-532-3p interacts with *FOXM1* 3’UTR, leading to decreased protein levels, thereby suppressing proliferation, migration, and invasion in colorectal cancer cells (Figure 4G).

## 3. Discussion

MicroRNAs, recognized as one of the smallest classes of biomolecules, exert dynamic regulatory influence on developmental conditions and diseases through their distinctive mechanisms [30,31,32]. Their impact spans various facets of mammalian development, including the neural system, placental site, and hematopoiesis, operating at the minutest scale [33,34,35,36]. In the context of cancer, microRNAs play a pivotal role in governing processes, ranging from the disease’s onset and proliferation to the regulation of metastasis, as well as influencing sensitivity or resistance to chemotherapeutic drugs [37,38,39,40,41]. Their involvement extends to shaping the tumor microenvironment as well [42]. MicroRNAs execute these multifaceted functions by targeting components of several signaling pathways crucial to both development and cancer [43,44,45].

In recent years, MicroRNA-532-3p has emerged as a tumor suppressor in various cancers. In CRC, it has been documented to disrupt Wnt/β-catenin signaling, effectively impeding the disease’s progression. The present study bolsters these findings and reveals the ability of miR-532-3p to upregulate p53 expression and to enhance cleavage in PARP. Notably, the ectopic overexpression of miR-532-3p in CRC cells resulted in a significant reduction in proliferative potential, migration, and invasion, corroborated by the previous study [20]. Similarly, its role in restraining proliferation and facilitating apoptosis has been investigated in lymphoma, where it targets β-catenin [21]. In prostate cancer, miR-532-3p hinders bone metastasis by inactivating NF-κB signaling [22]. Conversely, in renal cell carcinoma, clinical specimens showed a correlation between low expression levels of both mature strands of pre-miR-532, miR-532-5p, and miR-532-3p and the presence of the disease [23]. Despite its well-established tumor-suppressive role in most cancers, microRNA-532-3p, along with its other mature form, microRNA-532-5p, has been recognized for exhibiting oncogenic roles in hepatocellular carcinoma and breast cancer [25,28,29].

*FOXM1*, the new target of miR-532-3p that we discovered in this study, is a known master regulator of cell cycles [7]. This has made *FOXM1* a crucial factor in the occurrence and progression of cancer as a group of diseases that arises from dysregulation in a cell cycle [46,47]. As mentioned before, *FOXM1* has been of interest in reference to anticancer therapeutics as a key target [15,48]. Among the chemical inhibitors of *FOXM1*, thiazole antibiotics such as thiostrepton and siomycin A have been reported to induce apoptosis in human cancer cells by repressing the transcriptional activity of *FOXM1* [49,50]. Besides the cell cycle aspect, higher expression levels of *FOXM1* have been known to correlate with tumor invasion, leading to poor prognosis in colorectal cancer [11]. This ability of *FOXM1* to aid and promote tumorigenesis and metastasis has been studied in ovarian carcinoma, breast cancer, and non-small cell lung cancer as well [51,52,53]. These reports prove the importance of studying *FOXM1* and its regulation in the occurrence and progression of the disease.

In the present study, we have demonstrated the ability of microRNA-532-3p to suppress the proliferation, migration, and invasion of CRC cells. However, we did not observe a direct effect of miR-532-3p on cell death and the progression of a cell cycle. Furthermore, we established *FOXM1* as a novel target of miR-532-3p, adding another important link in the events leading to malignancy. This finding suggests that miR-532-3p could be a significant predictive marker for metastasis and prognosis in CRC. Further studies in other types of cancers where miR-532-3p has been reported as a tumor suppressor molecule will help gain a better insight of the picture.

This study validates the interaction between the microRNA-532-3p and 3’UTR of the *FOXM1* transcript, which results in decreased protein levels of *FOXM1*. This consequently suppressed the proliferation, migration, and invasion of colorectal cancer cells. The link between miR-532-3p and *FOXM1* could offer an insight on the direct tumor suppressive function exhibited by miR-532-3p in colorectal cancer, among other types of the disease.

## 4. Materials and Methods

### 4.1. Cell Lines and Culture

Human CRC cell lines (Caco2, Colo320, DLD1, HCT116, HT29, and SW480), and human embryonic kidney cells (HEK293T) (ATCC, Manassas, VA, USA) were grown in Dulbecco’s Modified Eagle’s Medium (DMEM), enriched with 10% fetal bovine serum (Gibco, Invitrogen, Waltham, MA, USA), and supplemented with antibiotics (100 X antibacterial–antimycotic solution, (GibCo, Grand Island, NY, USA). Cultures were maintained at 37 °C in a 5% CO_2_ atmosphere within a humidified chamber.

### 4.2. In Silico Mining of Databases and Selection of microRNAs

In the quest to identify microRNAs that may regulate the expression of *FOXM1* by interacting with its 3’UTR transcript, multiple databases were utilized. These databases fell into four distinct categories: predictive interaction (TargetScan, miRecords, and mirMap), network (Reactome and Biogrid), expression profile (TissueAtlas and dbDEMC 2.0), and literature (miRCancer). The results obtained from these searches were then subjected to Venn analysis to pinpoint the microRNAs that appeared most frequently. Subsequently, seven microRNAs were chosen from the compiled lists.

### 4.3. Cloning

The wild-type 3’UTR sequence and the sequences corresponding to the genes encoding the identified microRNAs were retrieved from the UCSC genome browser. Primers were designed, incorporating 100-base pair flanking regions for the specified genomic segments. Subsequently, amplicons were generated using platinum high-fidelity DNA polymerase (Thermo Invitrogen, Waltham, MA, USA). The obtained amplicons underwent restriction digestion: XhoI and NotI for the PsiCheck2 vector and *FOXM1* 3’UTR and BamHI and HindIII for the modified pRIP vector and microRNA amplicons. Ligation was performed at 4 °C utilizing the T4 ligase kit (Thermo Invitrogen, Waltham, MA, USA). The ligated products underwent validation for inserts through PCR, restriction digestion, and Sanger sequencing.

### 4.4. Dual-Luciferase Reporter Assay

A dual-luciferase reporter assay was performed as instructed in the manufacturer’s protocol (Promega, Madison, WI, USA). HEK293T cells were transiently co-transfected with PsiCheck2 vector containing *FOXM1* 3’UTR and a modified pRIP vector containing the gene coding for one of the selected microRNAs. After 72 h of transfection, the cells were lysed using Passive Lysis Buffer (1X). These lysates were used for the dual-luciferase reporter assay. A Promega Glomax luminometer was used to record the luminescence. An empty PsiCheck2 vector was used as the internal control.

### 4.5. Cell Proliferation and Colony Formation Assays

CRC cells (5 × 10^4^ cells/well) were plated in a 96-well cell culture plate and cultured at 37 °C with 5% CO_2_ in a humidified incubator (Thermo Scientific, Waltham, MA, USA) for 72 h. Cell survival was assessed using the MTT assay. MTT reagent was applied to each well and incubated in darkness at 37 °C for 4 h. Formazan crystals were then dissolved in isopropyl alcohol, and absorbance levels were measured. For the colony formation assay, cells (1 × 10^5^) were initially plated in 35 mm culture dishes and cultured in standard DMEM. Upon reaching approximately 40–50% confluency, cells underwent transfection with miR-532-3p and the control plasmid, employing Lipofectamine 3000 as per the manufacturer’s instructions. Transfection was halted after 24 h, followed by the addition of fresh media. Subsequently, cells were trypsinized and seeded at a density of 1000 cells per well in 6-well culture plates. The cells were then cultivated for a period of 14 days, with media renewal every 72 h. After the incubation period, cells were rinsed with PBS and fixed in 4% paraformaldehyde (PFA) for 5 min. Following PFA removal, the fixed cells underwent three washes with PBS. A 0.5% crystal violet solution was applied to the cells and allowed to incubate for 30 min at room temperature. The crystal violet solution was then aspirated, and the plate was washed under tap water before being air-dried. Subsequently, the colonies that formed were enumerated, with those containing 50 or more cells considered as positive.

### 4.6. Wound Healing and Cell Invasion Assays

Cells (5 × 10^4^ per well) were initially plated in a 12-well culture plate. Upon reaching 50% confluency, cells underwent transfection with miRNA-532-3p and the control plasmid as mentioned previously. Transfection was terminated after 24 h, and fresh regular media were introduced to the cells. When the cells reached approximately 90% confluency, a wound was generated by gently scratching with a sterile micropipette tip. Subsequently, the cells were examined under an inverted microscope every 24 h post washing with PBS.

For the matrigel invasion assay, CRC cells (1.5 × 10^5^) were seeded in 60 mm dishes and transfected with the control plasmid and miR-532-3p mimic. The cells were then incubated at 37 °C in OptiMEM. After 24 h, the transfected cells were trypsinized and diluted to a suspension of 5 × 10^4^ cells/mL. A 24-well plate with inserts containing a matrix (BD Biosciences, USA) was pre-wet with regular medium for two hours at 37 °C. Using sterile forceps, the inserts were carefully transferred onto these wells. The cell suspension was then added onto these inserts and incubated for 24 h. The invaded cells on the lower side of the membrane were stained with 0.5% crystal violet stain and counted in various fields.

### 4.7. Immunoblotting Analysis

The cells were harvested and rinsed with PBS, followed by lysis in RIPA buffer at 4 °C with agitation at 1400 RPM on a thermomixer for one hour and forty-five minutes. The resulting solution underwent centrifugation at 14,000 RPM for 15 min at 4 °C, and the supernatant, constituting whole-cell proteins, was collected. Subsequently, these whole-cell proteins were separated on a 10–12% polyacrylamide gel and transferred electrophoretically onto a polyvinylidene difluoride membrane at 100 V for two hours. After transfer, the membrane was blocked with a 5% *w*/*v* skimmed milk solution in tris-buffered saline with 0.5% tween-20 (TBST) for one hour at room temperature. The membrane was then incubated with various primary antibodies. Following this, the membranes were washed in TBST and subsequently probed with the appropriate secondary antibodies for 1 h at room temperature. Visualization was achieved using a chemiluminescence reagent (GE Healthcare Bio-Sciences, PA, USA) after washing the membrane with TBST. The following antibodies from various vendors were used for immunoblotting studies: anti-FOXM1 (Santa Cruz Biotechnology Inc. #sc-502, Dallas, TX, USA), anti-PCNA (Cat. #sc-7907, MA, USA) from Santa Cruz, Dallas, TX, USA, E-Cadherin (Cat. #3195, CST, USA), vimentin (Cat. #5741S, CST, USA), cleaved caspase 7 (Cat. #9492S, CST, USA), cyclin B1 (Cat. #4138S, CST, USA) GAPDH (Cat. #5174T, CST, USA), cleaved PARP (Cat. #9542S, CST, USA) from Cell signaling Technology Inc. (Beverly, MA, USA), and β-actin (Cat. #A5316, Sigma-Aldrich, St. Louis, MO, USA). The secondary antibodies used were anti-mouse-HRP (Cat #7076P2, CST, USA) and anti-rabbit HRP (Cat #ab6721, Abcam, Waltham, MA, USA).

### 4.8. RNA Isolation and Quantitative Real-Time PCR

Total RNA was extracted from CRC cell lines by using TRIzol (Invitrogen, Waltham, MA, USA). The Primescript RT reagent kit (Takara Biosciences, Westminster, CO, USA) was employed for the synthesis of single-strand cDNA. Real-time PCR detection was performed using SyBR TBgreen (Takara, Westminster, CO, USA). A specifically designed primer facilitated the synthesis of the stem-loop strand for miR-532-3p, which was subsequently detected using a specific forward primer and a universal reverse primer. RNU6B served as the internal control in this analysis. For the detection of transcripts from protein-coding genes, L19 was employed as the internal control. The quantification of expression fold change was determined using the 2^−ΔΔCt^ method.

### 4.9. Cell Cycle and Apoptosis Assays

For cell cycle analysis, after overexpressing with the miR-532-3p mimic and vector controls for 48 h, cells were washed with PBS, and fixed with 70% ethanol. Ethanol was added drop by drop while gently vortexing to ensure a uniform cell suspension, followed by incubation at 4 °C for one hour. Subsequently, the cells were washed and stained with propidium iodide solution, incubating for 10 min in the dark. The resulting suspension was passed through a sterile cell strainer and collected in a FACS tube. This suspension was then analyzed using the FACS ARIA machine. In case of apoptosis, the cells were harvested and washed with PBS. Then, they were resuspended in binding buffer, to which Annexin was added. This suspension was incubated in dark conditions at room temperature for 15 min. Then, 2 μL of propidium iodide solution was added to the suspension and it was incubated in the dark for 5 min. Furthermore, 400 μL of binding buffer was then added to this suspension, which was passed through a sterile cell strainer. This suspension was collected in an FACS tube and analyzed on an FACS ARIA machine.

### 4.10. Statistical Analysis

All experiments (apart from the matrigel invasion) were repeated three times independently. All data were expressed as mean ± SEM and analyzed with GraphPad Prism 8.0.2 software (GraphPad, Inc., La Jolla, CA, USA). Differences between two groups or more were analyzed by Student’s *t*-test or analysis of variance. *p* < 0.05 was considered to indicate statistically significant results.

## 5. Conclusions

This study validates the interaction between microRNA-532-3p and 3’UTR of *FOXM1* transcript, which resulted in decreased protein levels of *FOXM1*. This consequently suppressed the proliferation, migration and invasion in colorectal cancer cells. The link between miR-532-3p and *FOXM1* could offer an insight on the direct tumor suppressive function exhibited by miR-532-3p in colorectal cancer, among other types of the disease.

## Data Availability

The original contributions presented in the study are included in the article/Supplementary Materials; further inquiries can be directed to the corresponding author.

## Supplementary Materials

The following supporting information can be downloaded at: https://www.mdpi.com/article/10.3390/cancers16173061/s1. Table S1: Cloning primers used for amplifications of genomic regions for the selected microRNAs and *FOXM1* 3’UTR; Table S2: Primers used for quantitative real-time PCR of the selected genes and markers; Figure S1: Construct of the vectors used for cloning of the required microRNAs. Modified version of a pRIP vector that has the insertion region at the restriction sites of BamHI and HindIII enzymes, with a kanamycin selection marker; Figure S2: Confirmation of the plasmids with inserts of *FOXM1* 3’UTR and the selected microRNAs; Figure S3: Confirmation of clones by Sanger sequencing.
