# Supplementary material for: MicroRNA-532-3p Modulates Colorectal Cancer Cell Proliferation and Invasion via Suppression of FOXM1
Source: Cancers (Basel). 2024 Sep 2;16(17):3061. doi: 10.3390/cancers16173061 (PMC11394065; doi:10.3390/cancers16173061)
Supplement: Supplementary file 1 [file cancers-16-03061-s001.zip › cancers-3127326-supplementary.pdf]

Table S1: Cloning primers used for amplifications of genomic regions for the selected microRNAs and *FOXM1* 3'UTR. DSGene software was used.

No.	Target	Sequence of Primer	Annealing
1	**FOXM1 3-UTR F**	aaactcgagAAGCCTCTGAGTGAGGACAG	56°C
2	**FOXM1 3-UTR R**	aaagcggccgcGTCTCCATCAACACCCTCTTTC	
3	**miR149 pre F**	aaaggatccTTTGACTGCCGTGCGTCC	65°C
4	**miR149 pre R**	aaaaagcttTCAGCCACCTCTCACACCC	
5	**miR370 pre F**	aaaggatccAAGACAGAGAAGCCAGGTC	55°C
6	**miR370 pre R**	aaaaagcttGTTTCCCCATCAGCAAGATAG	
7	**miR423 pre F**	aaaggatccCGTACATTTTCCCGGATGG	55°C
8	**miR423 pre R**	aaaaagcttCTAAAACGAGAAGGAAGGGC	
9	**miR532 pre F**	aaaggatccACACAGTATACAGGAGAGCAG	55°C
10	**miR532 pre R**	aaaaagcttACAGTGTACCCCACATACAG	
11	**miR590 pre F**	aaaggatccCTTAAAAGTCCTGTAGCCAGTC	52°C
12	**miR590 pre R**	aaaaagcttGGGCAACTTCTATAACACCTG	
13	**miR671 pre F**	aaaggatccTCTACAACGCTGACTACCTG	59°C
14	**miR671 pre R**	aaaaagcttTGCTCCTGCTCAAAGAGAG	
15	**miR876 pre F**	aaaggatccTTCCTCAACTTTTGGTGGC	52°C
16	**miR876 pre R**	aaaaagcttTCAATGGCTCAGTAAAGGAATC	

Table S2. Primers used for quantitative real-time PCR of the selected genes and markers.

No.	Target	Primer sequence	Annealing
1	**RNU6B (U6) F**	5'AACGCTTCACGAATTTGCGT3'	60°C
2	**RNU6B (U6) R**	5'CTCGCTTCGGCAGCACA3'	
3	**miR-532 stem loop conversion primer**	5'GTCGTATCCAGTGCAGGGTCCGAGGTATTCGCACTGGATACGACTGCAAG3'	NA
3	**miR-532 F**	5'ATTAATGGAGGGTGTGGG3'	60°C
4	**Universal R**	5'CCAGTGCAGGGTCCGAGGTA3'	
5	**FOXM1 F**	5'GGCTCCCGCAGCATCAAGCA3'	60°C
6	**FOXM1 R**	5'AGGGTGGCCGCTCAGACACA3'	
7	**L19 F**	5'GCGGAGAGGGTACAGCCAAT3'	60°C
8	**L19 R**	5'GCAGCCGGCGCAAA3'	
9	**Vimentin F**	5'TGGCTAATTTGAGAGGTTCAGGT3'	60°C
10	**Vimentin R**	5'GGAGCACAGGAAGACTGCTA3'	
11	**Cyclin B1 F**	5'CCATTATTGATCGGTTCATGCA3'	60°C
12	**Cyclin B1 R**	5'CTAGTGGAGAATTCAGCTGTG3'	
13	**E-cadherin F**	5'GAAGGTGACAGAGCCTCTGGAT3'	60°C
14	**E-cadherin R**	5'GATCGGTTACCGTGATCAAAATC3'	

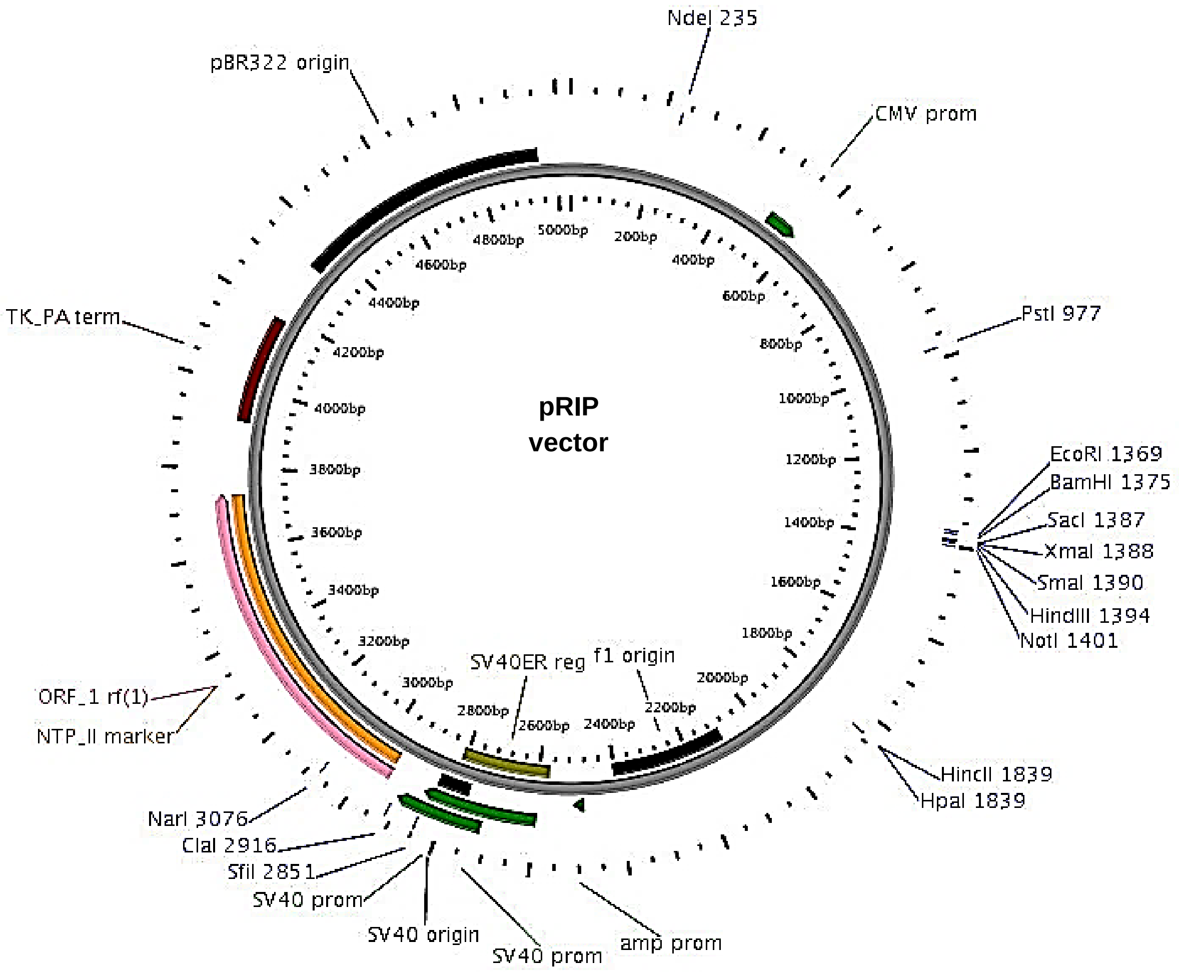

Figure S1. Construct of the vectors used for cloning of the required microRNAs. Modified version of a pRIP vector that has the insertion region at the restriction sites of BamHI and HindIII enzymes, with a kanamycin selection marker.

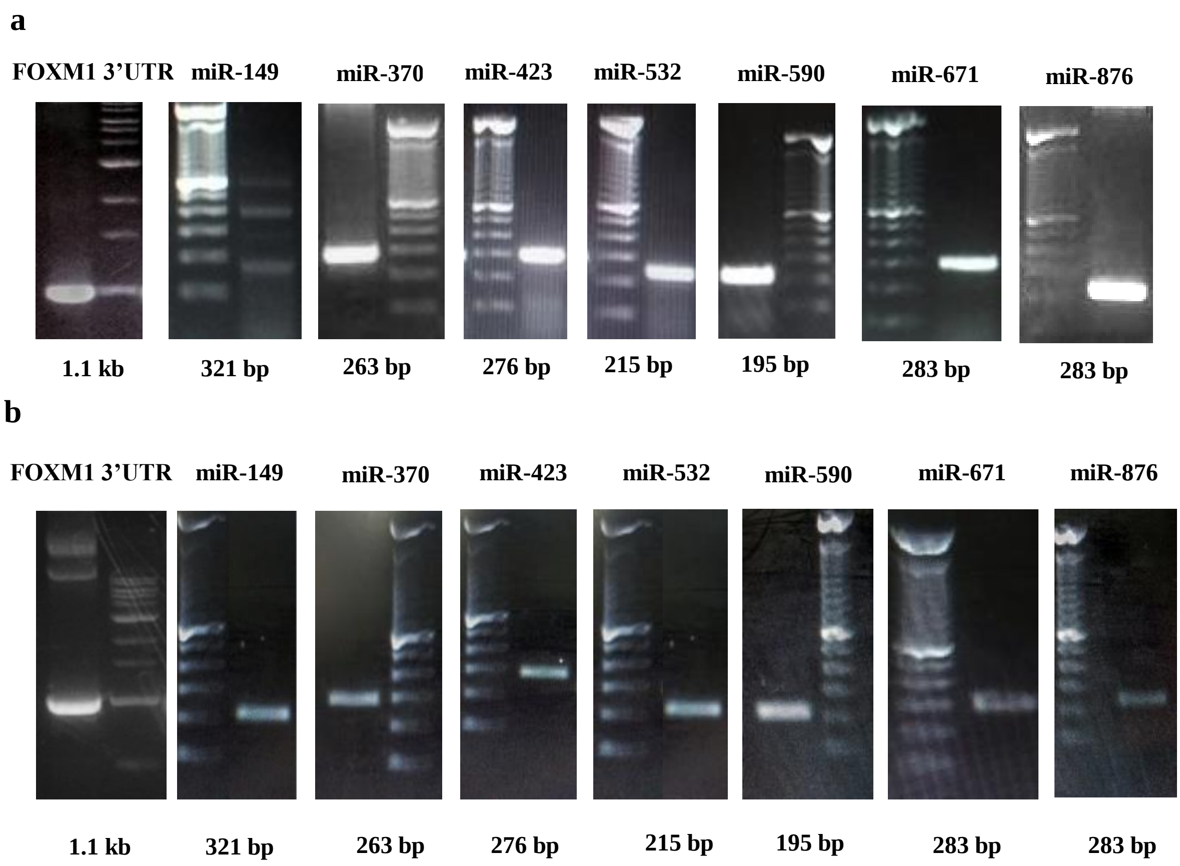

Figure S2. Confirmation of the plasmids with inserts of *FOXM1* 3’UTR and the selected microRNAs. Confirmation of the clones for *FOXM1* 3’UTR and selected microRNAs by **a.** PCR and **b.** restriction digestion using XhoI and NotI for *FOXM1* 3’UTR and BamHI and HindIII for the microRNA precursors.

a. MicroRNA-149

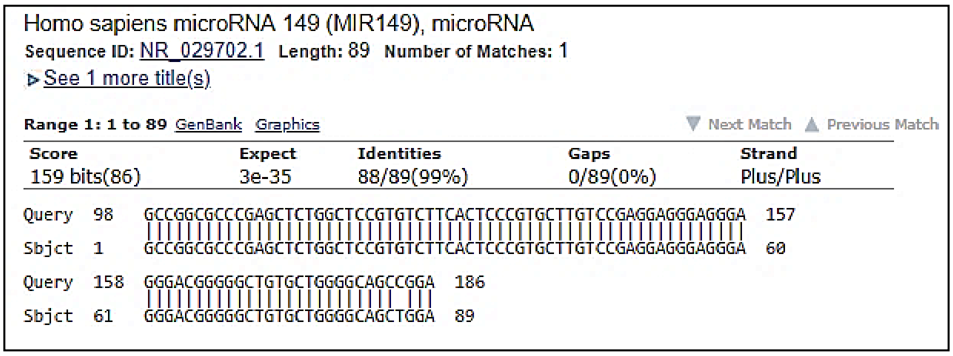

b. MicroRNA-370

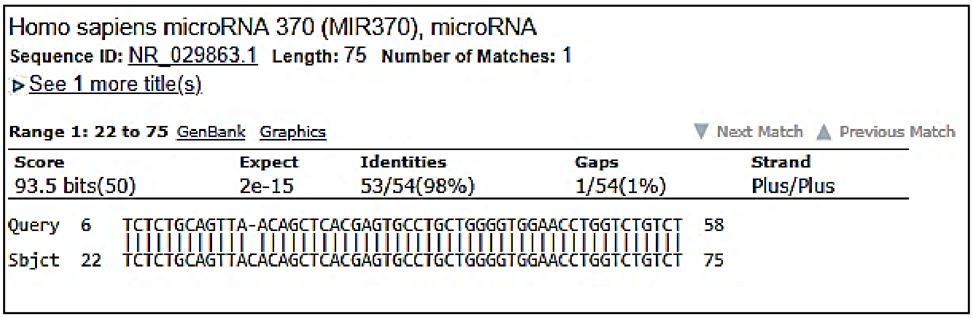

c. MicroRNA-423

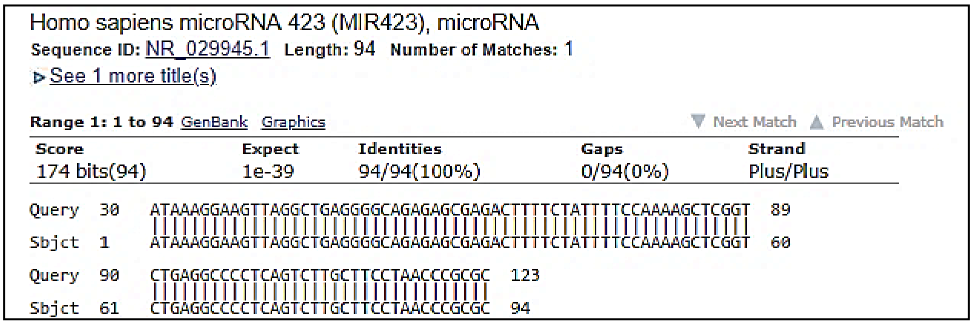

d. MicroRNA-532

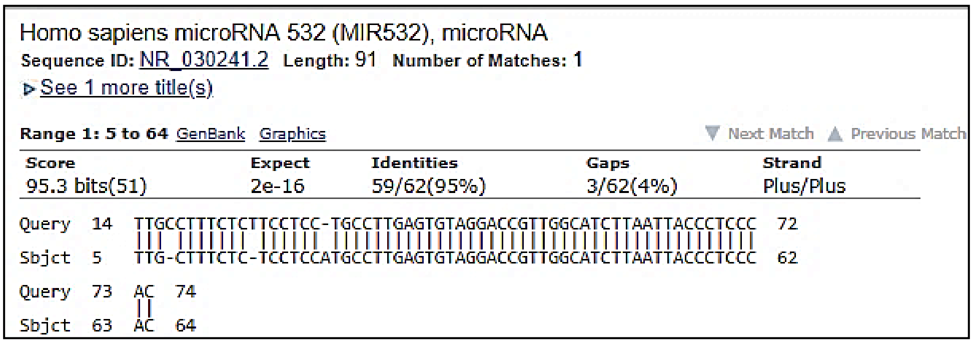

e. MicroRNA-590

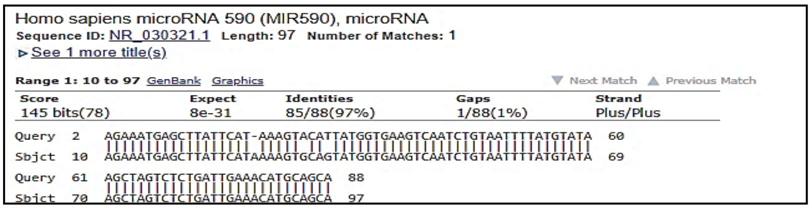

f. MicroRNA-671

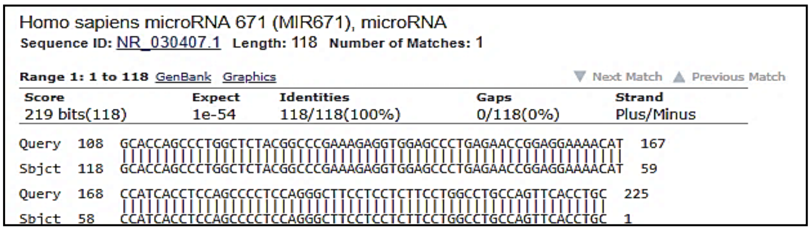

g. MicroRNA-876

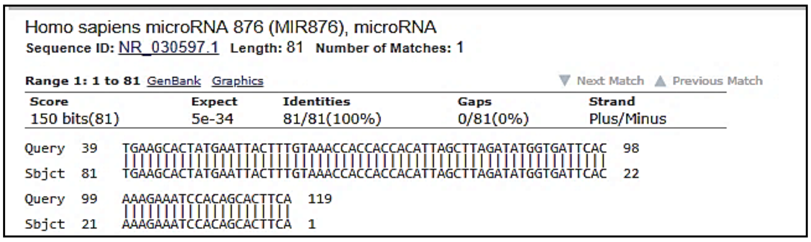

h. FOXM1 3'UTR

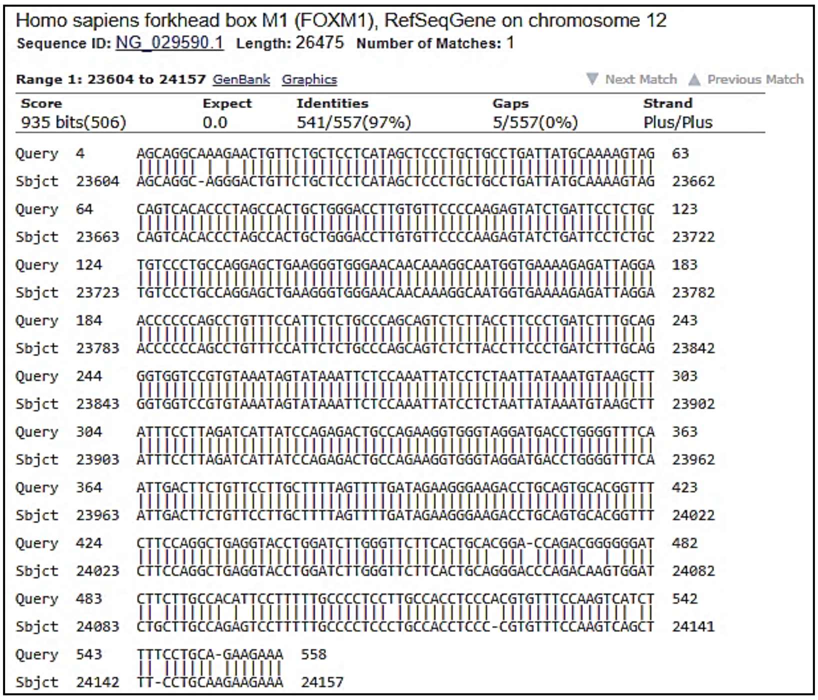

Figure S3. Confirmation of clones by Sanger sequencing
